# Urolithin B suppresses osteoclastogenesis via inhibiting RANKL‐induced signalling pathways and attenuating ROS activities

**DOI:** 10.1111/jcmm.17467

**Published:** 2022-07-03

**Authors:** Zechao Qu, Hao An, Mingzhe Feng, Wangli Huang, Dong Wang, Zhen Zhang, Liang Yan

**Affiliations:** ^1^ Department of Spine Surgery Honghui Hospital, Xi'an Jiao Tong University Xi'an China; ^2^ Medical College of Yan’an University Yan’an China; ^3^ Shaanxi University of Chinese Medicine Xian Yang China

**Keywords:** osteoclastogenesis, reactive oxygen species, signalling pathway, urolithin B

## Abstract

Osteoporosis (OP) has severely affected human health, which is characterized by abnormal differentiation of osteoclasts. Urolithin B (UB), as a potential natural drug, has been reported to exhibit numerous biological activities including antioxidant and anti‐inflammatory but its effects on OP, especially on RANKL‐stimulated osteoclast formation and activation, are still understood. In our study, we have demonstrated for the first time that UB inhibits RANKL‐induced osteoclast differentiation and explored its potential mechanisms of action. The RAW264.7 cells were cultured and induced with RANKL followed by UB treatment. Then, the effects of UB on mature osteoclast differentiation were evaluated by counting tartrate‐resistant acid phosphatase (TRAP)‐positive multinucleated cells and F‐actin ring analysis. Moreover, the effects of UB on RANKL‐induced reactive oxygen species (ROS) were measured by 2′, 7′‐dichlorodihydrofluorescein diacetate (DCFH‐DA) staining. Further, we explored the potential mechanisms of these downregulation effects by performing Western blotting and quantitative RT‐PCR examination. We found that UB represses osteoclastogenesis, F‐actin belts formation, osteoclast‐specific gene expressions and ROS activity in a time‐ and concentration‐dependent manner. Mechanistically, UB attenuates intracellular ROS levels by upregulation of Nrf2 and other ROS scavenging enzymes activation. Furthermore, UB also inhibited RANKL‐induced NF‐κB, MAPK and Akt signalling pathway and suppressed expression of c‐Fos and nuclear factor of activated T cells 1 (NFATc1), which is the master transcription factor of osteoclast differentiation. Taken together, our findings confirm that UB is a polyphenolic compound that can be a potential therapeutic treatment for osteoclast‐related bone diseases such as osteoporosis.

## 1 INTRODUCTION

Bone homeostasis is the primary metabolic process regulating bone structure and function during adult life maintained by the activity of osteoblasts and osteoclasts.^1^ Osteoclasts (OCs) are highly specialized multinucleated, giant cells that reabsorb bone tissue formed by the fusion of haematopoietic mononuclear progenitors of the monocyte/macrophage lineages.^2^ Abnormal differentiation or activities of osteoclasts can lead to various skeletal diseases such as Paget's disease of bone, periprosthetic osteolysis, bone tumours, osteoporosis and osteopetrosis.^3^ The activation of osteoclasts is regulated by systemic hormones and local cytokines produced either by marrow stromal cells, osteoblasts or T lymphocytes in the microenvironment.^3^, ^4^

It is well‐known that two indispensable molecules are vital for osteoclast survival, proliferation, differentiation and activation: macrophage‐colony stimulating factor (M‐CSF) and receptor activator of nuclear factor‐κB ligand (RANKL).^1^, ^5^, ^6^, ^7^, ^8^ RANKL, as the master regulator of osteoclastogenesis, is a member of the tumour necrosis factor (TNF) superfamily of cytokines that binds to its receptor activator of nuclear factor‐κB (RANK) expressed on osteoclast precursors to stimulate osteoclast differentiation, activation and survival.^9^, ^10^ Binding of RANKL to cell surface receptor RANK triggers the recruitment of tumour necrosis factor receptor‐associated factor 6 (TRAF6), resulting in the activation of downstream signalling pathways, including the mitogen‐activated protein kinase (MAPK), IκB/NF‐κB and PI3K/Akt pathways.^11^, ^12^, ^13^ The MAPK family contains p38, signal‐regulated kinase (ERK) and c‐jun N‐terminal kinase (JNK).^14^ Furthermore, these signalling cascade led to the induction and activation of transcription factors such as c‐Fos and nuclear factor of activated T cells 1 (NFATc1), the master transcription factor of osteoclast differentiation,^1^, ^6^, ^15^ ultimately, resulting in enhanced expression of osteoclast‐specific genes, tartrate‐resistant acid phosphatase (TRAP), cathepsin K (CTSK), matrix metallopeptidase‐9 (MMP‐9) and osteoclast stimulatory transmembrane protein (OC‐STAMP), which are associated with osteoclast differentiation and function.^16^

In addition, numerous studies have revealed that reactive oxygen species (ROS), as an intracellular signal mediator for RANKL‐induced osteoclasts formation and activation, has been testified to be a vital factor during bone remodelling and bone homeostasis, which promotes the differentiation of osteoclasts, accelerates bone resorption and contributes to a reduction of trabecular bone mass due to low levels of antioxidant enzymes.^17^, ^18^, ^19^, ^20^ Nuclear factor erythroid 2‐related factor 2 (Nrf2), as a redox‐sensitive transcription factor, plays a vital role in oxidative stress and inflammatory reactions and regulates the expression of different cytoprotective antioxidant enzymes such as haem oxygenase‐1 (HO‐1), catalase (CAT) and γ‐glutamyl cysteine synthetase catalytic subunit (GCLC), reducing the production of ROS and inhibiting RANKL‐mediated OCs.^21^, ^22^

Urolithin B (UB) is one of the most bioactive gut microbial metabolites of ellagitannins (ETs) that are polyphenolic compounds, enriched in diverse plant foods, such as pomegranates, berries, walnuts, tropical fruits and medicinal herbs.^23^, ^24^ It is well established that ETs are catabolized into ellagic acid (EA), which absorption is very low, and the compounds are further hydrolysed into urolithins (urolithin A–D) in the colon.^25^ Previously, UB has been reported to exhibit numerous biological activities including antioxidant, anti‐inflammatory, anti‐cancer, anti‐atherosclerotic and anti‐bacterial effects in LPS‐induced microglia and high glucose‐induced cardiomyocyte injury.^25^, ^26^, ^27^ Although several papers have been reported on biological activities of UB, the effects of UB on RANKL‐stimulated osteoclast formation have not been clearly demonstrated. Therefore, in this study, we deeply investigated the effects of UB on signalling pathway involved in osteoclastogenesis and to illuminate the involved precise molecular mechanisms in vitro.

## 2 MATERIALS AND METHODS

### 2.1 Reagents and antibodies

Urolithin B (purity ≥ 98%, CAS No.1139‐83‐9, molecular weight 212.2) was purchased from Med Chem Express and was dissolved in DMSO at the concentration of 1 mmol/L stock solution and stored in refrigerator in −80°C. Recombinant human RANKL was obtained from PeproTech EC, Ltd. Dulbecco's modified Eagle's medium (DMEM) and foetal bovine serum (FBS) were purchased from Gibco. The MTT assay kit was obtained from Roche. The TRAP staining kit was provided by Sigma‐Aldrich. Primary antibodies for c‐Fos and NFATc1 were purchased from Santa Cruz Biotechnology. Specific primary antibodies against phospho‐p38, p38, phospho‐ERK, ERK, phospho‐JNK, JNK, IκB, phospho‐NF‐κB p65, NF‐κB p65, Akt, phospho‐Akt, HO‐1, CAT and GCLC were purchased from Cell Signalling Technology. The primary antibody of Nrf2 was obtained from Thermo Fisher Scientific. β‐actin antibody was purchased from Sigma‐Aldrich, Inc.

### 2.2 OCs differentiation

RAW 264.7 mouse monocyte/macrophage cell lineage was obtained from American Type Culture Collection (ATCC). RAW264.7 cells were seeded in 96‐well plates and cultured in DMEM/10% FBS. Then, adherent cells on dish bottoms were cultured in α‐MEM supplemented with 10% FBS. They were treated with UB (during whole process of cell culture) at concentrations of 0, 10, 30, 50 and 100 μM, respectively. The cells were then stimulated with 40 ng/ml RANKL for 5 days. The medium was regularly replaced every 3 days. Osteoclasts could be identified by the multiple nuclei (≥ 3) and TRAP activity. Cell images were obtained using a digital camera attached to a Nikon ECLIPSE TE2000‐S microscope (Nikon).

### 2.3 Cell viability assays

RAW264.7 cells were seeded at a density of 3 × 10^3^ cells per well into 96‐well plates and stimulated with 40 ng/ml RANKL were treated with or without increasing concentrations of UB assess cytotoxicity for 2 and 5 days. Then, the 100 μl MTT solution (5 mg/ml) was added and incubated for 4 h. After aspirating the medium, 100 μl DMSO was added to each well, and the absorbance at 490 nm was measured using a 96‐well microplate reader (Tecan).

### 2.4 Measurement of intracellular ROS production

ROS assay kit (Beyotime) was used to detect the intracellular production of ROS based on the cell permeant fluorescent dye, 2, 7‐dichlorodihydrofluorescein diacetate (DCFH‐DA). Briefly, the RAW264.7 cells were seeded into a 96‐black well plate for 24 h. After pretreatment with the indicated concentrations of UB (0, 10, 50 and 100 μM) for 5 days, which already pretreated by 40 ng/ml RANKL. Then, they were tested by a fluorescent probe (1:1000) added DCFH‐DA in the dark and incubated at incubator for 30 min. The fluorescence of 2′, 7′‐dichlorofluorescein (DCF) was immediately measured using a fluorescence microscope. In addition, the mean fluorescence intensity was quantitative analysed using ImageJ software.

### 2.5 F‐Actin ring staining

To evaluate whether mature F‐actin ring formation was influenced by UB. We performed cytoskeletal fibrous actin (F‐actin) staining to observe sealing zone formation in RAW264.7 cells. Briefly, the cells were washed by PBS, after washing cells were fixed in 4% paraformaldehyde for 15 min, then cells washed by PBS three times, permeabilized with 0.1% Triton X‐100 for 15 min and stained with Rhodamine‐phalloidin in the dark for 1 h. For nuclear staining, cells were incubated with DAPI for 10 min. F‐actin stained cytoskeletons were visualized and quantified with a fluorescence microscope Nikon and nis‐Element analysis software (Nikon) for image analysis.

### 2.6 Western blotting analysis

RAW264.7 cells were seeded in 6‐well plates and incubated for 24 h. Cells were then pretreated with or without different concentrations of UB for 24 h followed by stimulation with 40 ng/ml RANKL for the indicated time. Cells were lysed in a buffer containing radio immune precipitation assay (RIPA) lysis buffer, PMSF and phosphatase inhibitors according to the manufacturer's instructions. The lysates were centrifuged at 4°C at 13,000 *g* for 15 min, and supernatants were collected. Quantitative analysis of protein concentration was performed using a BCA assay kit. Cellular proteins were loaded and separated by sodium dodecyl sulphate‐polyacrylamide gel electrophoresis (SDS‐PAGE). The proteins were then transferred to polyvinylidene difluoride (PVDF) membranes. The membranes were blocked with 5% skim milk for 2 h at room temperature and probed with primary antibodies at 4°C overnight. The membranes were washed in TBST three times and then incubated with a horseradish‐peroxidase (HRP)‐conjugated secondary antibody for 2 h. Finally, the signals of target proteins were detected with enhanced chemiluminescence reagent. The grey level of bands was analysed in ImageJ software.

### 2.7 Quantitative real‐time reverse transcription polymerase chain reaction (RT‐PCR)

RAW264.7 cells were seeded in a 6‐well plate, pretreated with different concentrations of UB for 2 h and then stimulated with RANKL (40 ng/ml) for 24 h. Total RNA was isolated from RAW264.7 cells using TRIzol (Thermo Fisher Scientific) according to the manufacturer's instructions RNA (1 μg) was reverse transcribed using oligo dT primers (10 μg) and dNTPs (10 mM). The obtained cDNA was used as a template for real‐time quantitative PCR on the LightCycler® 96 system. The reaction conditions of PCR amplification were as follows: denaturation at 95°C for 10 min and followed by 55 cycles at 95°C for 15 s, then decreased to 60°C for 15 s and finally increased to 72°C for 40 s. Primers employed for amplification were shown in Table 1. The housekeeping gene GAPDH was used as internal control.

**TABLE 1:** Primer sequences used for qRT‐PCR analysis

Gene name	Primer sequence (5′–3′) forward	Primer sequence (5′–3′) reverse
c‐Fos	5′–CGACCATGATGTTCTCGGGT–3′	5′–TCGGCTGGGGAATGGTAGTA–3′
NFATc1	5′–AGGACCCGGAGTTCGACTT–3′	5′–AGGTGACACTAGGGGACACA–3′
TRAP	5′–TGTCCGCTTGAGGGTACATT–3′	5′–GCAGGACAGCCCTTAGCATC–3′
MMP‐9	5′–CCAGCCGACTTTTGTGGTCT–3′	5′–CTTCTCTCCCATCATCTGGGC–3’
Cathepsin K	5′‐CTGGAGGGCCAACTCAAGAA–3′	5′–TGGCCCACATATGGGTAAGC–3′
OC‐STAMP	5′–CATCCGCTGCCTATTTGTGC–3′	5′–CACGCACATTGCCTAAGACG–3′

### 2.8 Immunofluorescence staining for p65 subunit

To investigate the nuclear translocation of NF‐κB p65 subunit, RAW264.7 cells were seeded in a 24‐well cover slips and pretreated with UB (100 μM) for 2 h before inducing them with RANKL (40 ng/ml) for 30 min. Cells were washed by PBS three times and fixed with 4% paraformaldehyde for 15 min at room temperature (RT). Then cells permeabilized with 0.1% Triton X‐100 for 15 min. Cells were incubated with rabbit primary antibody against NF‐κB p65 at 4°C overnight and then probed with Alexa Fluor 488 secondary antibody for 2 h in the dark at RT. Cover slips were washed with PBS for three times; cells were counterstained with DAPI for 15 min inspected using a confocal microscope (Nikon).

### 2.9 Statistical analysis

All data are expressed as the means ± SD for each group and were analysed by spss 24.0 software. One‐way analysis of variance (anova) in GraphPad Prism 7.0 was used to compare the differences between groups. The value of *p* < 0.05 was regarded as a statistically significant difference in our current study.

## 3 RESULTS

### 3.1 UB suppresses RANKL‐Induced OCs Differentiation without inducing cytotoxicity

**FIGURE 1:**
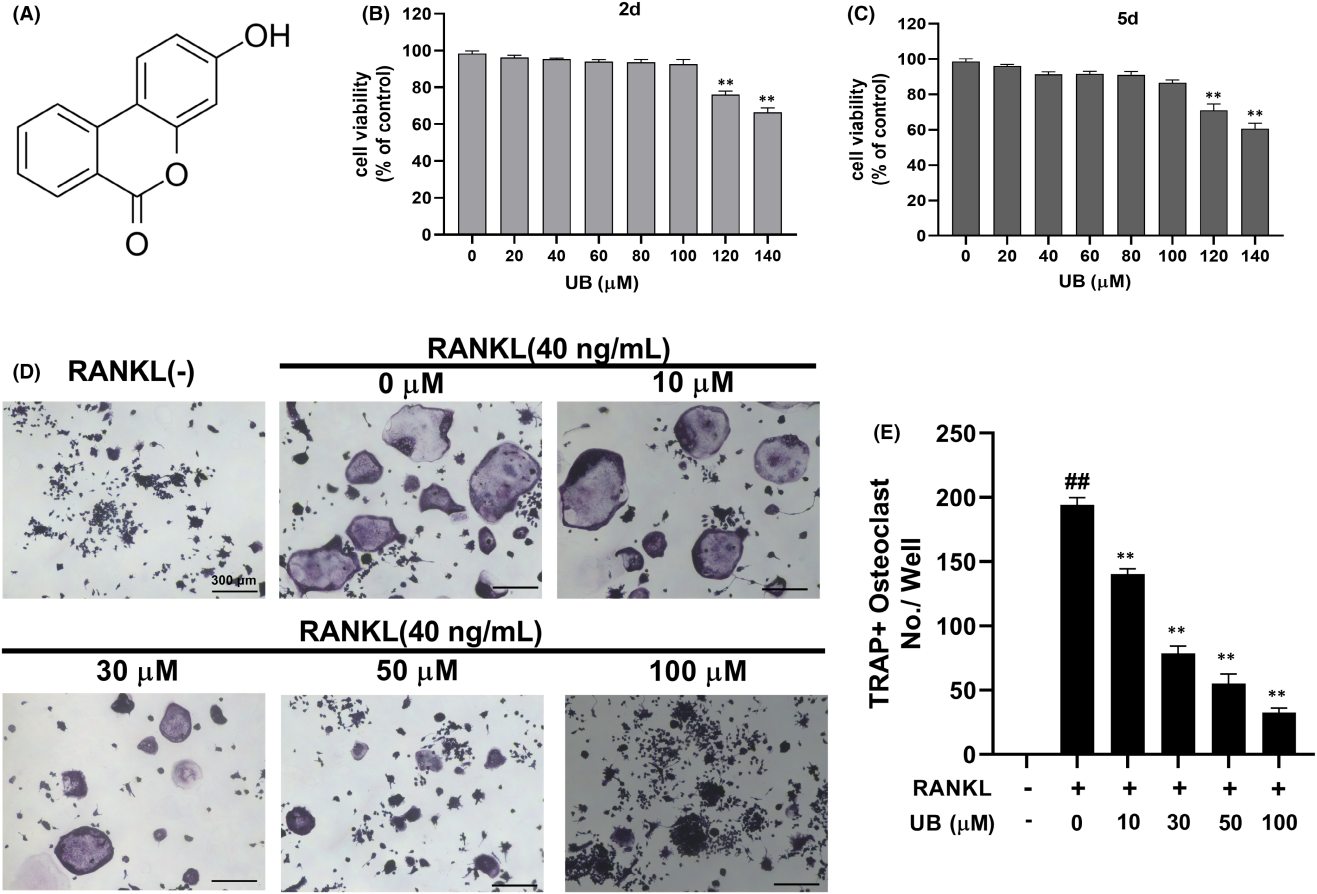
UB inhibits RANKL‐induced osteoclast formation and differentiation. (A) The chemical structure of Urolithin B (UB). (B, C) RAW264.7 cells were treated with different concentrations of UB (0, 20, 40, 60, 80, 100, 120 and 140 μM) for 2 and 5 days. Cell viability was measured by MTT assay. (D) RANKL‐stimulated RAW264.7 cells were treated with or without UB and stained with TRAP, and representative images are shown (Scale bar = 300 μm). (E) TRAP^+^ multinucleated (nuclei ≥3) cells in each treatment group were counted as mature osteoclasts. The above data are expressed as the mean ± SD of three independent experiments; ^##^ *p* < 0.01 vs. control group; * *p* < 0.05, ** *p* < 0.01 vs. RANKL‐induced group

To detect the effects of UB (Figure 1A) on RANKL‐induced OCs Differentiation. RAW264.7 cells were treated with various concentrations of UB in the presence of RANKL (40 ng/ml). Results showed that numerous TRAP‐positive multinucleated osteoclasts were formed in the RANKL‐induced control group, while increasing concentrations of UB inhibited TRAP‐positive multinucleated osteoclast formation in a dose‐dependent manner. To examine the effect of UB treatment on cell viability during RANKL stimulation, cell viability was then evaluated for 2 and 5 days. As shown in (Figure 1B, C) the treatment of UB for cells with the concentrations <100 μM did not affect the viability of cells. The results demonstrated that the inhibitory effect of UB on OCs differentiation was not due to cellular cytotoxicity.

### 3.2 UB down‐regulates osteoclast differentiation and function

**FIGURE 2:**
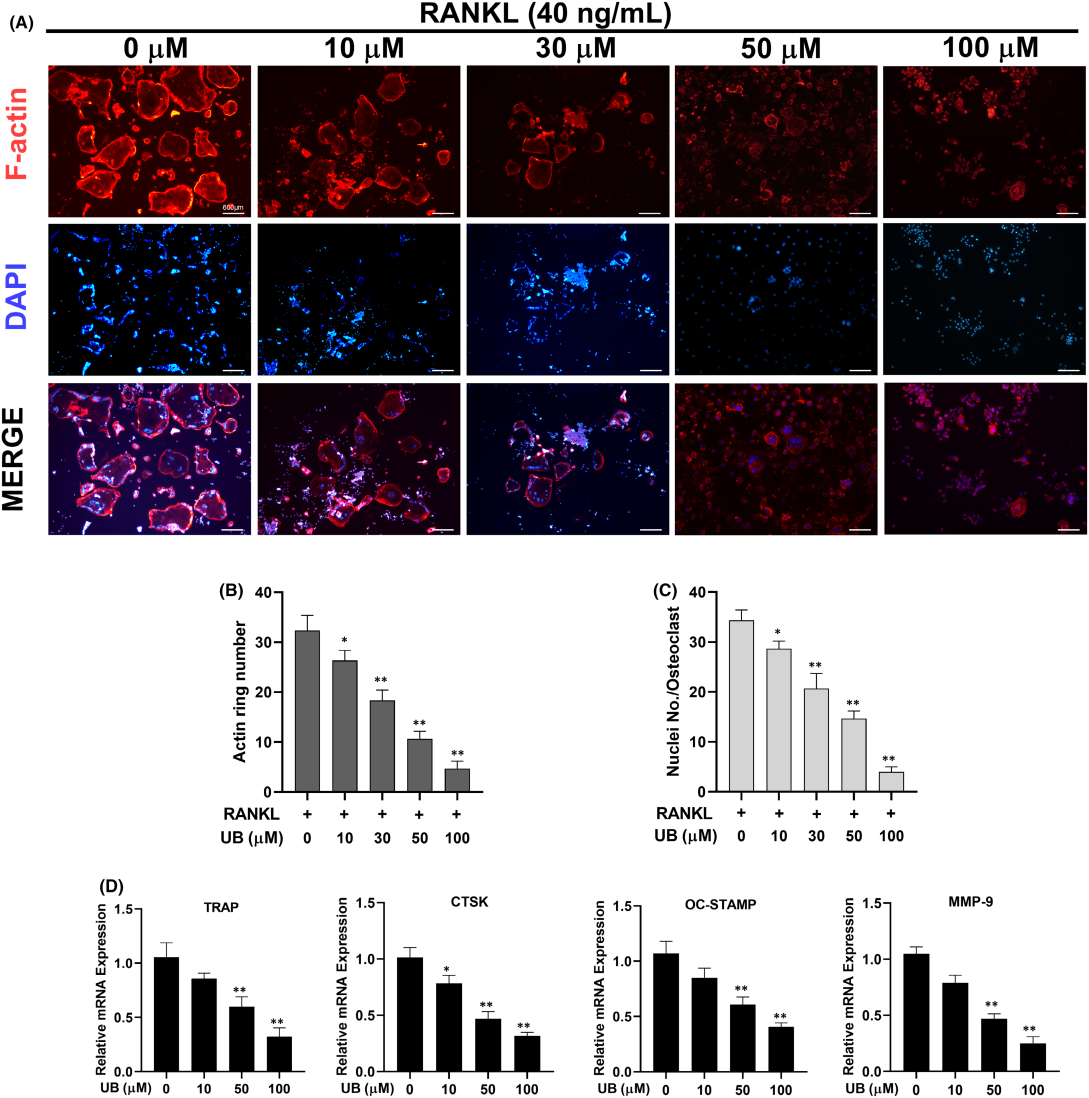
UB affects F‐Actin ring formation and inhibits osteoclast‐specific genes expression. (A) RAW264.7 cells were stimulated with RANKL in the presence or absence of UB (0, 10, 30, 50 and 100 μM) for the indicated concentrations. F‐Actin (red) and nuclei (blue) images were obtained by fluorescence microscope (Scale bar = 600 μm). (B, C) Quantification analyses of F‐Actin size and nucleus number per osteoclast. (D) The expressed mRNA levels of TRAP, cathepsin K, MMP‐9 and OC‐STAMP were quantitatively measured by qRT‐PCR in the absence or presence of different concentrations of UB. Data are expressed as the means ± SD of three independent experiments; * *p* < 0.05, ** *p* < 0.01 vs. RANKL group

Document studies have shown that TRAP staining has been identified as one of the most significant and credible experimental methods to recognize osteoclasts and their precursors. At present, TRAP positive and nuclear number ≥3 can be recognized as osteoclasts. From the results of TRAP staining, increasing concentrations of UB distinctly reduced the size and number of osteoclast formations in a dose‐dependent manner (Figure 1D, E). Furthermore, cytoskeletal structure F‐actin formation is one of the critical functional areas in mature osteoclasts. Mature osteoclasts were determined by assessing the osteoclastic cytoskeletal sealing zone structure formation after stimulation by RANKL. The number of osteoclasts and nuclei was observed by rhodamine‐phalloidin staining. However, our current results showed that after treatment with UB, both number and morphology of F‐actin rings were down‐regulated in a dose‐dependent manner (Figure 2A). In particular, F‐actin ring formation was significantly inhibited at the concentration of 100 μmol/L compared to that of other groups (Figure 2B, C).

### 3.3 UB reduces RANKL‐induced mRNA expression of osteoclast‐specific genes

To further elucidate the role of UB in osteoclast differentiation, quantitative PCR (qPCR) was used to analyse and quantify the mRNA expression levels of RANKL‐induced osteoclast‐associated genes (including TRAP, cathepsin K, MMP‐9 and OC‐STAMP) in the absence or presence of UB. These gene activation regulates osteoclastogenesis and up‐regulate OC differentiation. We examined these genes at mRNA level using qPCR to observe how UB affected osteoclast‐specific genes expression. However, these genes were strongly suppressed following the addition of UB in a dose‐dependent manner (Figure 2D). These results further revealed that osteoclastogenesis markedly suppressed by UB treatment.

### 3.4 UB attenuates the RANKL‐activated NF‐κB，MAPK and Akt signalling pathway

**FIGURE 3:**
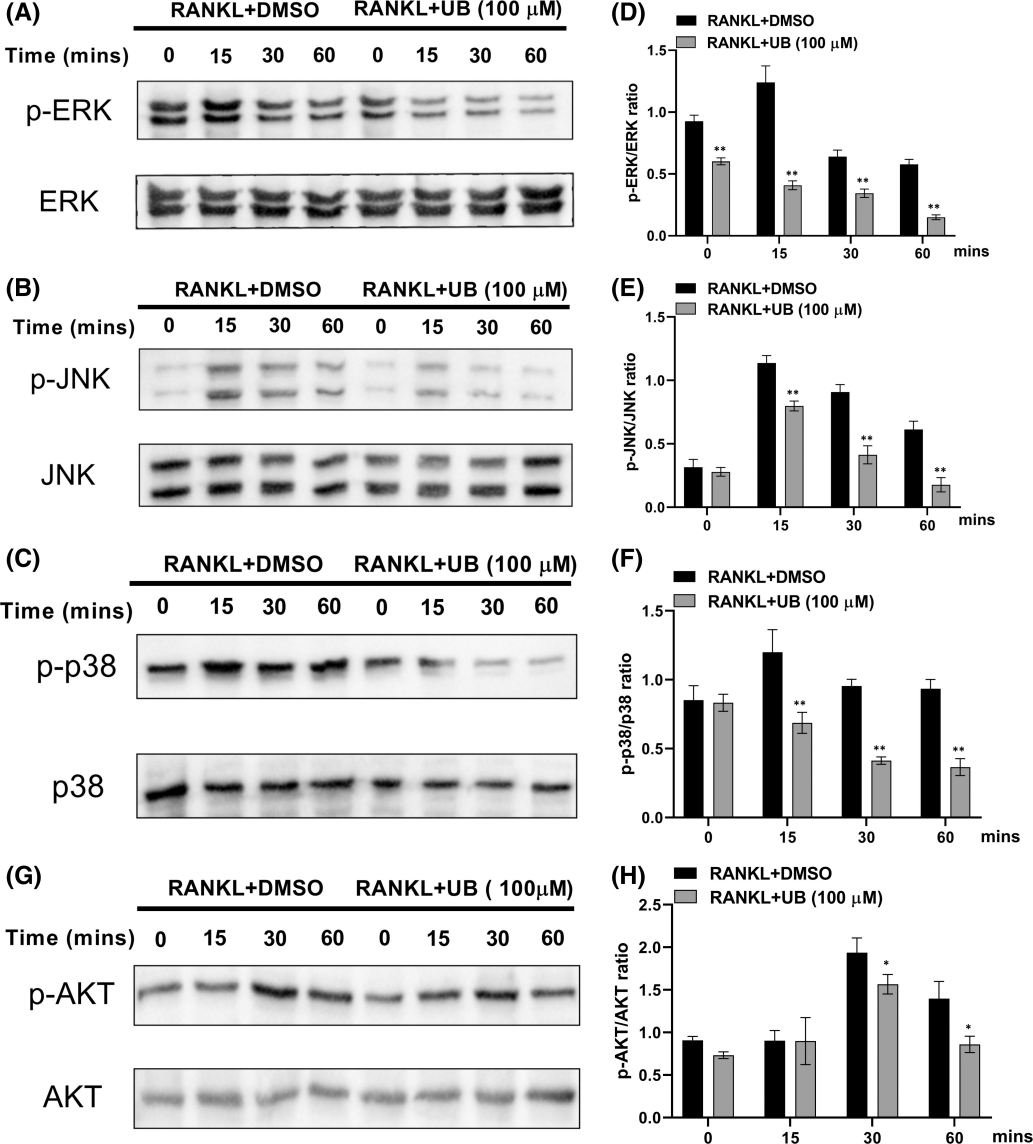
UB inhibits RANKL induced MAPK and Akt signalling pathways. Raw264.7 cells were pretreated with UB for 4 h, followed by 40 ng/ml RANKL for the indicated times; then, total proteins were extracted and evaluated by Western blot assays for MAPKs (A–C) and Akt (G) dependent pathways. Average ratio of phosphor‐ERK relative to ERK, phosphor‐JNK relative to JNK, phosphor‐p38 relative to p38, and phosphor‐Akt relative to Akt were shown. The bands' intensity analysis (D–E, H) was quantified by image J software. Data are expressed as the means ± standard deviation of three independent experiments; *, *p* < 0.05, **, *p* < 0.01 vs. RANKL+DMSO group

**FIGURE 4:**
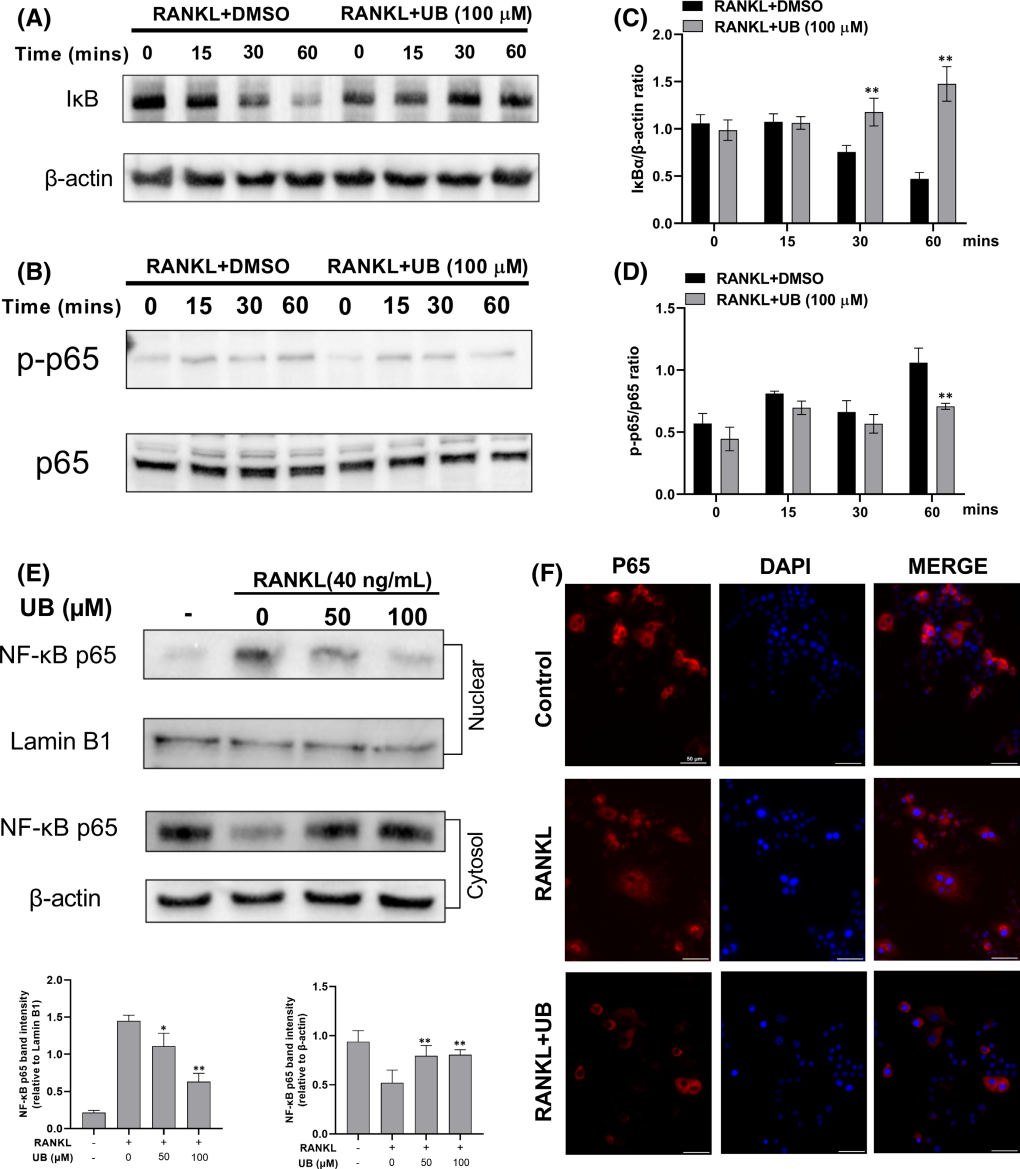
Effects of UB on RANKL‐activated IκB/NF‐κB signalling pathway. (A, B) RAW264.7 cells were preincubated with UB for 4 h and then treated with 40 ng/ml RANKL at 0, 15, 30 and 60 min relative to the non‐UB treatment (0 μM). Total protein was extracted and subjected to Western blot analysis using antibodies against NF‐κB p‐p65, p65, IκB and β‐Actin. (C, D) The relative intensities of the protein bands were normalized to β‐Actin and analysed by image J software. (E) RAW264.7 cells were pretreated with UB for 2 h prior to RANKL (40 ng/ml) stimulation for 30 min. Then, whole cytoplasmic and nuclear proteins were extracted and subjected to Western blot analysis using antibodies against NF‐κB p65. The levels of β‐Actin and Lamin B1 expression were measured to confirm equal protein amounts in cytoplasmic fractions and nuclear extracts. (F) RAW264.7 cells were pretreated with UB (100 μM) for 2 h and then stimulated with 40 ng/ml RANKL for 30 min. Immunofluorescence staining (Scale bar = 50 μm) was performed to locate NF‐κB p65 (red). Nuclear counter staining was performed using DAPI (blue). Data are expressed as the means ± standard deviation of three independent experiments; *, *p* < 0.05, **, *p* < 0.01 vs. RANKL+DMSO group

The NF‐κB, MAPK (ERK, p38 and JNK) and Akt both play critical roles in RANKL‐induced osteoclastogenesis. Therefore, to determine how UB influences NF‐κB, MAPK (p38, ERK and JNK) and Akt signalling pathways during osteoclast differentiation, we detected the protein expression levels of p‐JNK, p‐ERK, p‐P38, p‐Akt, I‐κB and p‐NF‐κB p65 at 0, 15, 30 and 60 min after 40 ng/ml RANKL stimulation without or with UB (100 μM). The results indicated that UB markedly suppressed phosphorylation of NF‐κB p65, Akt, MAPK (p38, ERK and JNK) and degradation of IκB of pre‐osteoclasts under stimulating by RANKL (Figures 3, 4). These results demonstrated the inhibitory capacity of UB on NF‐κB, MAPK (ERK, p38 and JNK) and Akt phosphorylation within the osteoclasts. For the MAPK signalling pathway, as shown in (Figure 3A–F), phosphorylation of ERK (Figure 3A), p38 (Figure 3C) and JNK (Figure 3B) relative to total ERK, total p38 and total JNK was suppressed significantly by UB treatment in RAW264.7 cells. Furthermore, translocation of free P65 into the nucleus of NF‐κB signalling pathway is crucial for RNAKL‐induced OCs activation. To further determine the role of RANKL‐induced nuclear translocation of NF‐κB p65 in the inhibitory effect of UB, we performed Western blotting and immunofluorescence staining. As shown in Figure 4E, RANKL stimulation markedly promoted the translocation of cytosolic p65 into the nuclear, while pretreated with UB significantly blocked the RANKL‐induced free p65 nuclear translocation in a dose‐dependent manner. Further fluorescent signal confirmed pretreated with UB for 2 h suppressed p65 nuclear translocation and caused more p65 to be retained in the cytoplasm (Figure 4F).

### 3.5 UB prevents c‐Fos and NFATc1 expression in osteoclast differentiation

**FIGURE 5:**
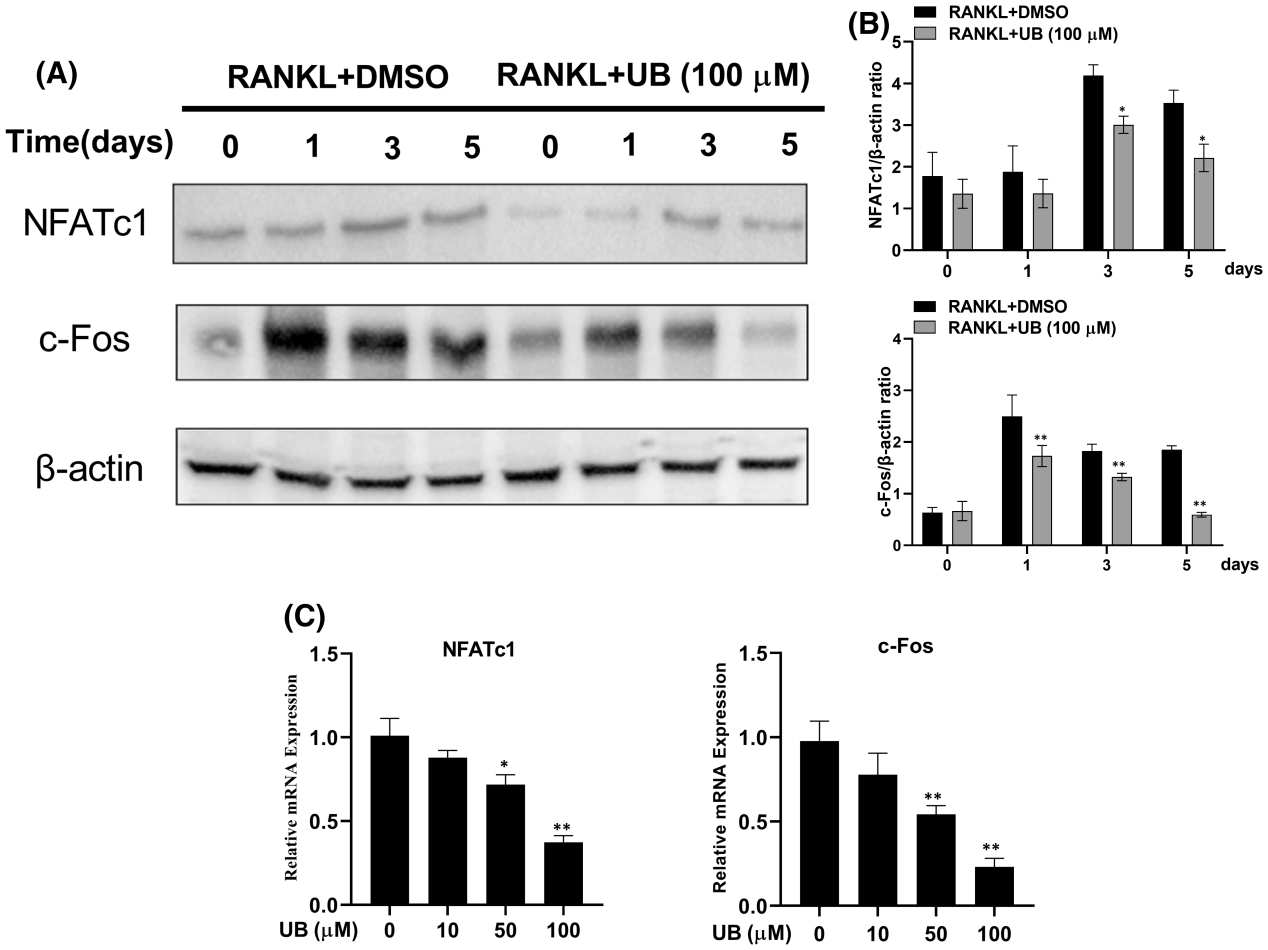
Effect of UB on RANKL‐induced the OCs specific mRNA and protein expression of c‐Fos and NFATc1. (A) RAW264.7 cells were treated with or without UB (100 μM) on Day one, Day three and Day five, and the proteins including c‐Fos and NFATc1 were measured using Western blot. (B) The expression of protein bands was quantitatively analysed related to β‐Actin and calculated by image J software. (C) Quantitative analyses of relative mRNA expression of OCs specific genes: Transcription factors NFATc1 and c‐Fos were performed by real‐time RT‐PCR in the RAW264.7 cells induced with RANKL and RANKL+ indicated concentrations of UB. All data in the figures are presented as mean ± standard deviation of three independent experiments; *p < 0.05, **p < 0.01, vs. non‐UB treatment (0 μM)

As c‐Fos and NFATc1 are the master transcription factors of osteoclast differentiation, we investigated whether UB could suppress c‐Fos and NFATc1 expression in the presence of RANKL via real‐time PCR and immunoblotting assay. RAW264.7 cells were pretreated with UB and further cultured with RANKL for 0, 1, 3 and 5 days. As shown in (Figure 5), stimulation with RANKL gradually enhanced the mRNA and protein levels of c‐Fos and NFATc1 in a time‐dependent manner. However, our current study results showed the expression of c‐Fos and NFATc1 at the mRNA (Figure 5C) and protein levels (Figure 5A) were significantly inhibited by the UB group. UB significantly attenuated the proteins including c‐Fos and NFATc1 mainly on Days three and five (Figure 5B). Taken together, these results proved that the inhibitory effects of UB may also be involved in the inhibition of RANKL‐induced c‐Fos and NFATc1 expression, thus effectively promoting the anti‐osteoclast effect of UB.

### 3.6 UB decreases RANKL‐induced ROS production in osteoclastogenesis via the upregulation of Nrf2 and other antioxidant enzymes

**FIGURE 6:**
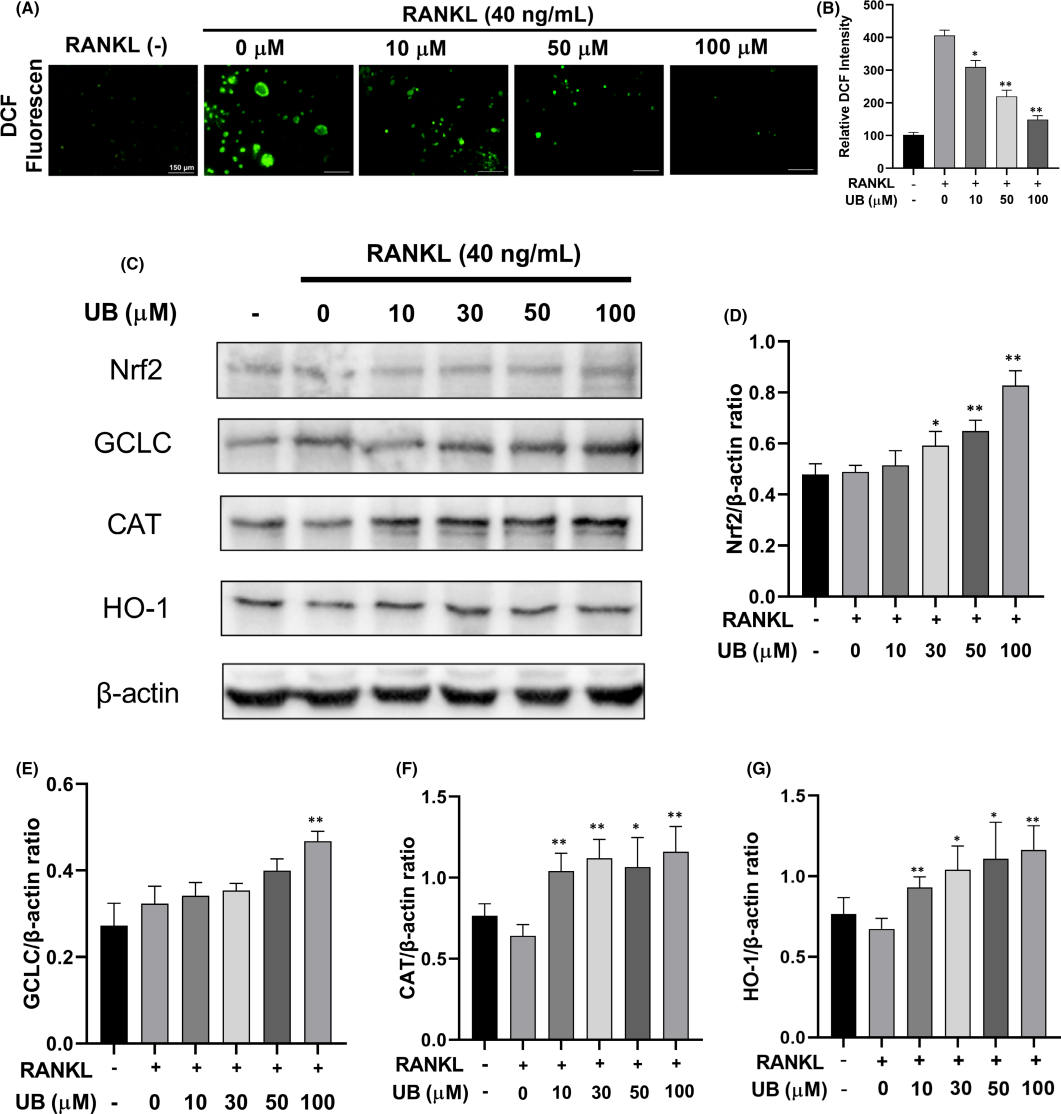
UB reduces RANKL‐induced ROS production on osteoclastogenesis by enhancing the expression of Nrf2 and increasing ROS scavenging enzymes expression. (A) Representative confocal images of RANKL‐induced ROS generation in RAW264.7 cells with or without pretreatment of UB at different concentrations, and then, intracellular ROS was detected by DCFH‐DA (Scale bar = 150 μm). (B) Quantification of relative DCF fluorescence intensity averaged on cells of each well were evaluated and quantified. (C) Representative Western Blot images show the effects of UB on the expression of Nrf2 and several antioxidant enzymes including GCLC, catalase and HO‐1 that regulate the production of ROS. RAW264.7 cells were stimulated by RANKL (40 ng/ml) in the absence or presence of UB (0, 10, 30, 50, 100 μM) for 2 days, and then, proteins were harvested for Western blot. (D–G) Quantification of the ratios of band intensity of GCLC, catalase and HO‐1 relative to β‐Actin. All data are expressed as the means ± SD of at least three independent experiments; * *p* < 0.05, ** *p* < 0.01 vs. RANKL‐induced group

It has been proved that RANKL‐mediated intracellular ROS production plays a crucial role during osteoclast formation.^18^ Therefore, we investigated the production of ROS during osteoclastogenesis using DCFH‐DA staining. As shown in (Figure 6A), UB treatment markedly reduced intracellular ROS production in a dose‐dependent manner compared with the RANKL treatment alone. The intensity of DCF fluorescence was also significantly lower in the presence of UB (Figure 6B). DCFH‐DA staining indicated that UB could effectively inhibit the RANKL‐induced ROS production during the development of osteoclastogenesis. Furthermore, Nrf2 has been considered a major contributing factor in oxidative stress and inflammatory reactions, which regulates the expression of several antioxidant enzymes (HO‐1, CAT and GCLC) and reduces the production of ROS.^28^ Thus, we assessed UB's effect on ROS‐related enzymes including Nrf2, HO‐1, CAT and GCLC expression via Western blot. As shown in (Figure 6C–G), the Western blot results showed that the expression of antioxidant enzymes was reduced by RANKL stimulation but was recovered and enhanced dose dependently by UB treatment. Taken together, these data mechanistically indicated that UB abrogated RANKL‐induced intracellular ROS production through the upregulation of Nrf2 and other ROS‐scavenging enzymes.

## 4 DISCUSSION

In life, bone is a kind of dynamic yet stable connective tissue that is made of cellular fibres and matrix, removed by osteoclasts and formed by osteoblasts.^29^ However, dysregulation of bone homeostasis results in various bone diseases, especially osteoporosis. The main pathological cause is the excessive absorption and differentiation of osteoclasts, which leads to the reduction of bone mass and the destruction of bone structure.^30^ Clinical representative anti‐osteoporosis drugs mainly include bisphosphonates, oestrogens, serotonin and calcitonin, but there are many restrictions and side effects such as increased risk of breast cancer, osteonecrosis of the jaw, allergies, the risk of venous thromboembolism and neurological reactions.^31^, ^32^ Previous study proved RANKL, as the master regulator of osteoclastogenesis, has been a potential valuable target for the treatment of pathological bone loss.^7^, ^33^ Document studies have been reported that UB as a polyphenolic compound has antioxidant, anti‐inflammatory, anti‐cancer, anti‐atherosclerotic and anti‐bacterial effects.^23^, ^25^ Previous researchers have identified that UB has a significant ability to suppress the NF‐κB and Akt signalling pathway activation.^34^ In this study, our results have demonstrated for the first time that UB not only inhibited osteoclastogenesis by repressing MAPK signalling pathway, NF‐κB signalling pathway, Akt signalling pathway, c‐Fos and NFATc1, but also effectively by regulating the expression of antioxidant enzymes such as CAT, GCLC and HO‐1, decreased intracellular ROS generation during osteoclastogenesis. Therefore, UB can be a new treatment option for RANKL‐induced osteoclast differentiation.

Firstly, to assess the biological function of UB, an osteoclast differentiation assay was carried out. After UB stimulation, RANKL‐induced osteoclast differentiation and function were gradually inhibited in a dose‐dependent manner. The fused multinuclear cells form a large well‐polarized F‐actin ring is one of the key organelles for osteoclast motility and bone resorption functions.^35^, ^36^ As shown in our study, UB interferes with the podosome belt formation, which further verified the inhibitory effect of UB on osteoclast formation. At the molecular level, our current study results showed that the inhibitory effect was achieved by inhibiting the expression levels of osteoclast‐specific genes (TRAP, CTSK, MMP‐9, OC‐STAMP).

It is well‐known that NF‐κB plays a pivotal role in RANKL‐induced OCs differentiation.^37^ The classic NF‐κB signalling pathway involves the activation of the inhibitor of κB (IκB) kinase (IKK) complex, phosphorylates IκBα and then free P65 would translocate into the nucleus to initiate OC‐related genes transcription.^7^, ^38^ Therefore, the inhibition of degraded IκBα and free P65 are critical to inhibit OCs formation and bone resorption. Our results showed that treatment with UB reduced the cytoplasmic degradation of IκBα and decreased RANKL‐stimulated p65 phosphorylation and the nuclear translocation of P65. This finding indicates that the inhibitory capacity of UB on OCs formation could be concerned with the NF‐κB signalling pathway (Figure 4).

Moreover, MAPK signalling pathway has been regarded as a crucial downstream factor in RANKL‐stimulated OCs differentiation.^39^ As an important transmitter of signals from the cell surface to the interior of the nucleus, MAPK family members (p38, ERK and JNK) regulate many vital cellular physiological/pathological processes, such as cell growth, differentiation, stress adaptation to the environment and inflammatory responses.^40^, ^41^ MAPK signalling pathway plays an important role in RANKL‐mediated OCs growth, differentiation and apoptosis. Among them, p38 regulates the early‐stage differentiation of osteoclasts by upgrading the activity of microphthalmia‐associated transcription factor (MITF) and TRAP expression.^42^, ^43^ ERK1/2 and JNK1/2 are essential in the proliferation and differentiation of osteoclasts, and the expression of p‐ERK1/2 and p‐JNK1/2 can induce OCs proliferation and differentiation.^7^, ^44^ Besides, PI3K/Akt pathway is involved in a variety of cellular functions in the formation of osteoclasts, including mitosis, survival, motility and differentiation. In the current study, the results demonstrated that compared with the control group, the phosphorylation level of RANKL‐mediated p38, JNK1/2, ERK1/2 and Akt in the UB treatment groups suppressed significantly in a dose‐dependent manner (Figure 3).

According to previous reports, the lack of c‐Fos expression results in a differentiation block in the osteoclast lineage and c‐Fos^−/−^ mice presented severe osteopetrosis because of the absence of mature OCs.^15^, ^45^ Another report states that osteoclast‐specific conditional NFATc1‐deficient mice develop osteopetrosis due to impaired osteoclastogenesis.^45^ These results clearly indicate that NFATc1 and c‐Fos are two indispensable and essential transcriptional factors during the process of osteoclastogenesis in vitro and in vivo.^46^ In this study, we found that after Raw264.7 cells were treated with 40 ng/mL RANKL, with or without 100 μM UB for 0, 1, 3 or 5 days. The results of Western blotting suggest that UB could also negatively affect the expression levels of NFATc1 and c‐Fos (Figure 5).

Documented studies demonstrated that RANKL‐induced ROS production plays an important role in the development of osteoporosis.^18^ Normal metabolism of the body can produce ROS, which is mainly produced by mitochondrion electron transport chains and contains superoxide anion radical, hydrogen peroxide, hydroxyl radical and nitric oxide. Under oxidative stress, Nrf2 detaches from Keap1 and translocates to the nucleus, which controls the expression of antioxidant enzymes including CAT, GCLC and HO‐1.^20^, ^47^ It has been previously reported that UB could exhibit antioxidant and anti‐inflammatory biological activities and promote Nrf2 nuclear translocation against superoxide production and apoptotic cell death during hypoxia/reoxygenation insult.^23^, ^25^ We investigated the effect of UB on intracellular oxidative stress during its inhibition upon stimulation with RANKL, and it resulted in that UB significantly suppressed intracellular ROS levels. Moreover, we demonstrated that several ROS scavengers (CAT, GCLC and HO‐1) were determined to be upregulated by UB to abrogated osteoclast differentiation in this study. RANKL stimulation reduced the gene expression of Nrf2 and was beneficial to ROS signalling,^48^ whereas UB treatment recovered and increased Nrf2 expression dose dependently, suggesting that UB may abrogate intracellular ROS at least in part via the upregulation of Nrf2 and other antioxidant enzymes (Figure 6).

**FIGURE 7:**
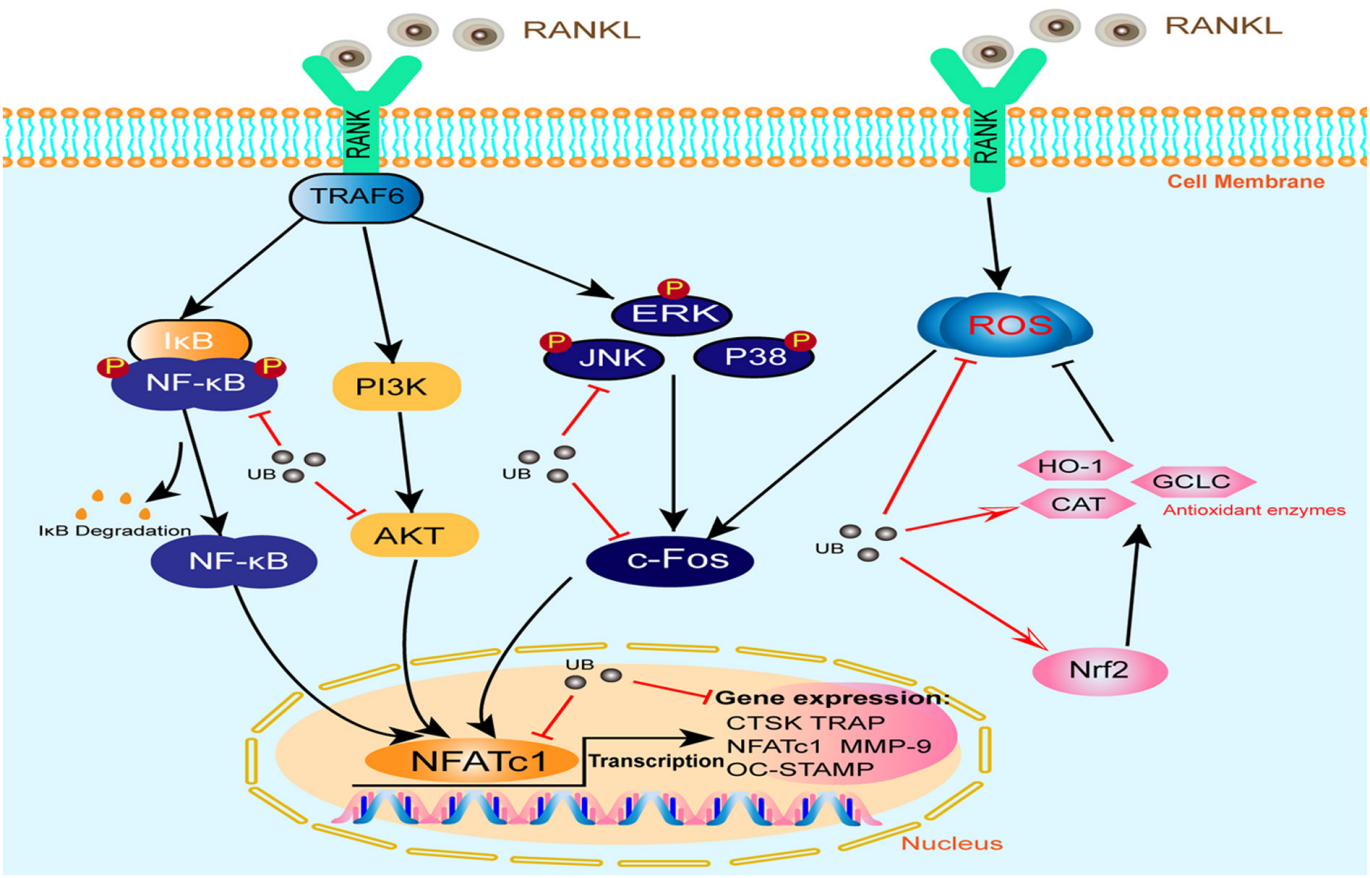
Proposed scheme of UB restrains osteoclastogenesis by inhibiting RANKL‐induced signalling pathways and attenuating ROS activities. Upon RANKL binding to RANK, both NF‐κB and MAPK pathways are activated, contributing to the c‐Fos and NFATc1 self‐amplification and translocation into nucleus. As a result, several osteoclast‐specific genes (TRAP, CTSK, MMP‐9 and OC‐STAMP) are upregulated. Likewise, ROS production is enhanced by the stimulation of RANKL. Our results for the first time demonstrated that UB could attenuate osteoclastogenesis, possibly via abrogating the activation of MAPKs, AKT and NF‐κB signalling pathways and can suppress intracellular ROS level, further impairs downstream activation of key signal cascades such as c‐Fos and NFATc1 transcription factors. RANKL, receptor activator of nuclear factor‐κB ligand; NF‐κB, nuclear factor‐κB; MAPK, mitogen‐activated protein kinases; NFATc1, nuclear factor of activated T cells 1; c‐Fos, Proto‐oncogene c‐Fos; ROS, reactive oxygen species; HO‐1, haem oxygenase‐1; CAT, catalase; GCLC, γ‐glutamyl cysteine synthetase catalytic subunit; UB, Urolithin B; CTSK, cathepsin K; MMP‐9, matrix metalloproteinase‐9; TRAP, tartrate‐resistant acid phosphatase; OC‐STAMP, osteoclast stimulatory transmembrane protein

In summary, our results for the first time proved that UB could attenuate osteoclastogenesis, possibly via abrogating the activation of MAPKs, Akt and NF‐κB signalling pathways and can suppress intracellular ROS level by inhibiting RANKL‐induced ROS production and activating expression of intracellular antioxidant enzyme, which thereby impairs the downstream activation of key signalling cascades including c‐Fos and NFATc1 transcription factors (Figure 7). However, future research is required to further explore the effect of UB on osteoporosis and osteolysis in vivo. Meanwhile, the mechanisms investigated in the current study should also be further conducted. Taken together, our findings may provide a cellular and molecular mechanism for UB as a promising candidate medication on RANKL‐mediated osteoclast differentiation and bone loss diseases, such as osteoporosis and osteolysis.

## AUTHOR CONTRIBUTIONS

**zechao qu:** Conceptualization (equal); data curation (equal); formal analysis (equal); resources (equal); software (equal); validation (equal); writing – original draft (equal). **Hao An:** Conceptualization (equal); data curation (equal); formal analysis (equal); software (equal). **mingzhe feng:** Investigation (equal); methodology (equal). **wangli huang:** Methodology (equal). **dong wang:** Investigation (equal); methodology (equal). **Zhen Zhang:** Validation (equal); writing – review and editing (equal). **liang yan:** Methodology (equal); project administration (equal); supervision (equal); validation (equal).

## CONFLICT OF INTEREST

The authors confirm that there are no conflicts of interest.

## CONSENT FOR PUBLICATION

The manuscript is approved by all authors for publication.

## Data Availability

All data and materials were included in the manuscript.
